# Maternal hyperuricemia and adverse maternal-fetal outcomes: a systematic review and meta-analysis of observational studies

**DOI:** 10.3389/fmed.2026.1704136

**Published:** 2026-03-09

**Authors:** Ahmed Abu-Zaid, Khawlah Habib AlShammari, Sara N. Alenezi, Reem Mohammad, Fatemah Sayer Althaher, Mohammad Murad, Fajer Ali Alkandari, Manar M. Alshammari, Abdullah M. Alharran, Saeed Baradwan, Mohammed Abuzaid, Osama Alomar

**Affiliations:** 1 Department of Biochemistry and Molecular Medicine, College of Medicine, Alfaisal University, Riyadh, Saudi Arabia; 2 Kuwait Institute for Medical Specializations, Kuwait City, Kuwait; 3 Royal College of Surgeons in Ireland, Dublin, Ireland; 4 College of Medicine and Medical Sciences, Arabian Gulf University, Manama, Bahrain; 5 College of Pharmacy, University of Sharjah, Sharjah, United Arab Emirates; 6 Department of Obstetrics and Gynecology, King Faisal Specialist Hospital and Research Center, Jeddah, Saudi Arabia; 7 Department of Obstetrics and Gynecology, Al Birk General Hospital, Al Birk, Saudi Arabia; 8 Department of Obstetrics and Gynecology, King Faisal Specialist Hospital and Research Center, Riyadh, Saudi Arabia; 9 Department of Obstetrics and Gynecology, College of Medicine, Alfaisal University, Riyadh, Saudi Arabia

**Keywords:** hyperuricemia, preeclampsia, pregnancy, preterm birth, uric acid

## Abstract

**Background:**

Maternal serum uric acid (SUA) has been suggested as a biomarker for adverse pregnancy outcomes, but findings remain inconsistent. This systematic review and meta-analysis evaluated the association between elevated maternal SUA levels and key obstetric and neonatal outcomes.

**Methods:**

A comprehensive search of PubMed, Scopus, Web of Science, and the Cochrane Library was conducted through December 2024. Observational studies reporting associations between high maternal SUA levels and pregnancy outcomes were included. Data were pooled using a random-effects model as odds ratios (ORs). Heterogeneity was assessed using the *I*^2^ statistic.

**Results:**

A total of 30 studies met the inclusion criteria. Elevated maternal SUA levels were significantly associated with increased odds of preterm birth (PTB; OR = 2.05, 95% CI: 1.55–2.72, *I*^2^ = 90.22%, *n* = 20), preeclampsia (PE; OR = 3.84, 95% CI: 2.17–6.77, *I*^2^ = 92.35%, *n* = 8), neonatal intensive care unit (NICU) admission (OR = 2.20, 95% CI: 1.63–2.97, *I*^2^ = 0.00%, *n* = 5), cesarean delivery (OR = 1.44, 95% CI: 1.16–1.79, *I*^2^ = 92.59%, *n* = 8), and intrauterine growth restriction (IUGR; OR = 3.03, 95% CI: 1.16–7.91, *I*^2^ = 84.23%, *n* = 8). Elevated SUA levels were also associated with low Appearance, Pulse, Grimace, Activity, and Respiration scores at 1 min (OR = 3.63, 95% CI: 1.47–8.95, *I*^2^ = 62.75%, *n* = 4) and 5 min (OR = 4.66, 95% CI: 2.45–8.85, *I*^2^ = 0%, *n* = 4). Conversely, high SUA levels were associated with reduced odds of spontaneous vaginal delivery (SVD; OR = 0.68, 95% CI: 0.51–0.91, *I*^2^ = 93.29%, *n* = 8) and a non-significant reduction in live birth (OR = 0.65, 95% CI: 0.41–1.02, *I*^2^ = 55.98%, *n* = 4).

**Conclusion:**

This meta-analysis shows an association between elevated maternal SUA levels and adverse maternal and neonatal outcomes. However, the evidence is derived from heterogeneous observational studies and does not support causal inference or routine SUA-based screening in clinical practice. Maternal SUA may be a promising biomarker, but large, well-designed prospective studies are needed to validate these findings and clarify its incremental predictive value.

**Systematic review registration:**

CRD420251038421, https://www.crd.york.ac.uk/PROSPERO/view/CRD420251038421.

## Introduction

Hyperuricemia, characterized by an abnormally high concentration of uric acid in the blood, affects a substantial proportion of the adult population worldwide (1), with prevalence ranging from 5 to 25% depending on factors such as age, sex, and the presence of comorbid conditions (2). During pregnancy, serum uric acid (SUA) levels are physiologically expected to decline in early gestation and then rise slightly in later stages; however, persistently elevated levels may indicate underlying pathophysiological disturbances (3, 4).

In the context of pregnancy, elevated SUA levels have gained attention due to their potential association with adverse maternal and neonatal outcomes. Conditions such as low Apgar scores, intrauterine growth restriction (IUGR), preterm birth (PTB), and preeclampsia (PE) remain leading causes of morbidity and mortality, particularly in low- and middle-income countries (5, 6). Identifying reliable biomarkers to predict these outcomes continues to be a critical clinical priority (7, 8).

Recent studies have suggested that elevated maternal SUA levels may be associated with a range of adverse obstetric outcomes (9, 10). Hyperuricemia has been associated with impaired placental development, increased oxidative stress, endothelial dysfunction, and inflammation, which are key mechanisms implicated in pregnancy complications such as PE, IUGR, and PTB (11, 12). Several observational studies have suggested that uric acid may serve as a prognostic biomarker in high-risk pregnancies, particularly for hypertensive disorders (13–15). However, inconsistent findings, variations in the definition of hyperuricemia, and heterogeneous study designs have made it difficult to draw firm conclusions about the strength and direction of these associations.

While individual studies have evaluated the relationship between maternal hyperuricemia and pregnancy complications, findings have been inconsistent, and no universally accepted cutoff values have been established for elevated uric acid in pregnancy (16, 17). Previous reviews have often focused on specific outcomes, such as PE, without providing a comprehensive synthesis of the broader obstetric implications (12, 16, 18). Moreover, prior studies have shown considerable variability in methodological quality, population characteristics, and the timing of SUA measurement, which has hindered interpretation and generalizability. To date, there is a lack of comprehensive and methodologically rigorous synthesis of observational evidence examining the association between elevated maternal SUA levels and a wide range of pregnancy outcomes.

Given uric acid’s potential as an accessible and cost-effective biomarker for assessing pregnancy-related risks and the current lack of consensus in the literature, a systematic review and meta-analysis is warranted. The present study aimed to assess and quantify the association between maternal hyperuricemia and multiple adverse pregnancy outcomes, such as PE, PTB, low Apgar scores, and neonatal intensive care unit (NICU) admissions. By synthesizing available evidence from observational studies, this review aimed to clarify the clinical relevance of maternal uric acid levels in prenatal care and guide future research on risk stratification and early intervention strategies.

## Methods

### Study protocol

This systematic review was designed, executed, and reported in accordance with the Preferred Reporting Items for Systematic Reviews and Meta-Analyses (PRISMA) guidelines (19). A detailed protocol was prospectively registered with the International Prospective Register of Systematic Reviews (PROSPERO; CRD420251038421). All primary outcomes and main meta-analyses were pre-specified in the registered study protocol.

### Search strategy

A comprehensive and systematic search strategy was implemented to identify relevant studies from the inception up to December 2024 of databases including PubMed/Medline, Scopus, Web of Science, and the Cochrane CENTRAL Library. The search strategy combined both keywords and Medical Subject Headings (MeSH) and was carefully tailored to the unique indexing systems of each database to optimize retrieval efficiency. The primary objective of the literature search was to identify studies investigating the association between SUA levels and a range of pregnancy outcomes. A broad spectrum of outcome-related terms was used, including but not limited to “Pregnancy Outcome,” “Preterm Birth,” “Stillbirth,” “Intrauterine Fetal Death (IUFD),” “Preeclampsia,” “Neonatal Intensive Care Unit (NICU) admission,” “Cesarean delivery,” “Intrauterine Growth Restriction (IUGR),” “APGAR score,” “spontaneous vaginal delivery (SVD),” and “Live birth.” These were paired with exposure-related terms such as “Uric Acid,” “Urate,” “Trioxopurine,” “Hyperuricemia,” and variations such as “Hyperuri*” and “Hypouri*” to ensure sensitivity and inclusivity across platforms. The search strategy was inclusive of all languages, provided the terms appeared in English, and was developed in collaboration with a professional information scientist to ensure its accuracy and comprehensiveness. In addition to database searches, manual screening of reference lists from selected articles and relevant reviews was performed to identify any studies that might have been overlooked during the electronic search process. Efforts were also made to include unpublished studies to reduce the risk of publication bias. A complete list of the search terms and strategy details is available in Supplementary File 1.

### Study eligibility criteria

To be eligible for inclusion in the meta-analysis, studies were required to meet several predefined criteria to ensure methodological rigor, relevance, and sufficient data availability. Specifically, (i) only primary research articles involving direct data collection from human participants were included, as meta-analyses rely on original numerical data to calculate pooled effect sizes. Additionally, (ii) studies had to focus on pregnant women aged 19 years or older, and (iii) they were required to examine the association between maternal serum uric acid levels and various pregnancy or neonatal outcomes. (iv) The outcomes of interest were pre-specified and harmonized across studies prior to quantitative synthesis. These included PTB, PE, NICU admission, live birth, mode of delivery [spontaneous vaginal delivery (SVD) vs. cesarean delivery], fetal growth restriction outcomes [small for gestational age (SGA) or IUGR], and low Apgar scores at 1 and 5 min. When outcome definitions differed across studies (e.g., SGA vs. IUGR, Apgar score at 1 vs. 5 min), the outcomes were only grouped if the definitions were deemed clinically and methodologically equivalent; otherwise, they were analyzed separately or excluded from the pooling.

Furthermore, (v) observational study designs, including cohort, case–control, and cross-sectional studies, were considered eligible. To ensure data quality, (vi) studies had to report confirmed patient data, include relevant demographic information, and provide measurements of serum uric acid levels during pregnancy. Finally, (vii) studies were included only if they reported at least one of the specified pregnancy or neonatal outcomes, ensuring alignment with the objectives of the review.

Studies were excluded if (i) they involved animal subjects, (ii) they lacked original data, such as reviews, commentaries, or editorials, (iii) they were case reports or case series, or (iv) the abstract or full text could not be retrieved despite extensive efforts using Internet searches, academic library networks, and direct contact with corresponding authors.

### Data extraction and quality assessment

A comprehensive and standardized approach was used to extract, manage, and evaluate data from each included study, using pre-tested coding manuals and customized Excel-based worksheets designed to accommodate specific study designs, such as cohort or case–control studies. The extracted study characteristics included key information, including first author’s name, publication year, sample size, country of origin, maternal age, study design, and the operational definitions used for outcomes. Outcome harmonization was conducted prior to meta-analysis by mapping study-specific definitions onto standardized outcome categories. Only studies using comparable definitions were pooled within the same meta-analytic model. When the outcome definitions were non-equivalent or insufficiently comparable, quantitative pooling was avoided. For quantitative synthesis, we primarily extracted raw, unadjusted data, e.g., number of events and non-events or crude effect estimates, and used these data to calculate pooled effect sizes. Adjusted effect estimates were not pooled, even when reported, because of substantial variability in statistical models, covariate selection, and definitions of confounders across studies, e.g., age, BMI, parity, hypertensive disorders of pregnancy, gestational diabetes, and renal function. Where reported, information on adjustment methods and covariates was extracted descriptively and summarized qualitatively. This approach was adopted to ensure methodological consistency and comparability across studies, thereby minimizing bias arising from heterogeneous adjustment strategies.

To ensure consistency and minimize bias, data collection was conducted independently by three groups of reviewers, each consisting of two authors. Each group screened a portion of the studies and extracted relevant data based on the inclusion criteria. In cases of disagreement within or between groups regarding study eligibility or data extraction, the issue was resolved through consultation with a third, independent reviewer to reach consensus.

Furthermore, the methodological quality of each study was assessed using the Newcastle–Ottawa Scale (20), which evaluates studies based on criteria related to selection, comparability, and outcome assessment. This tool, encompassing seven items, allowed reviewers to classify studies into categories of low, medium, or high risk of bias based on a scoring system ranging from 1 to 9. Disagreements during the quality assessment process were resolved by consensus between the reviewers. This rigorous process ensured a systematic, transparent, and reliable evaluation of study quality and data integrity throughout the review.

### Data analysis

Meta-analysis was considered only if at least three included studies reported sufficient data on the association between SUA levels during pregnancy and maternal or neonatal outcomes. Unadjusted odds ratios (ORs) were pooled using a random-effects model (21) based on data from studies reporting the outcomes of interest. This meta-analysis was conducted using Stata version 18.0 (StataCorp LLC, College Station, TX, United States). Heterogeneity was assessed using chi-squared (Q) and *I*^2^ statistics. In accordance with established methodological recommendations, visual inspection of funnel plots, Egger’s regression test, and Begg’s rank correlation test were used to assess small-study effects and publication bias in meta-analyses with 10 or more studies (*k* ≥ 10). We also performed a meta-regression and subgroup analysis based on SUA cutoff levels, trimester of sampling, and maternal age for variables over five included studies. To assess the influence of each individual study on the overall effect size, a sensitivity analysis was also performed. The certainty of the evidence for each meta-analyzed variable was evaluated using the Grading of Recommendations Assessment, Development, and Evaluation (GRADE) approach (22).

## Results

### Study selection

**Figure 1:**
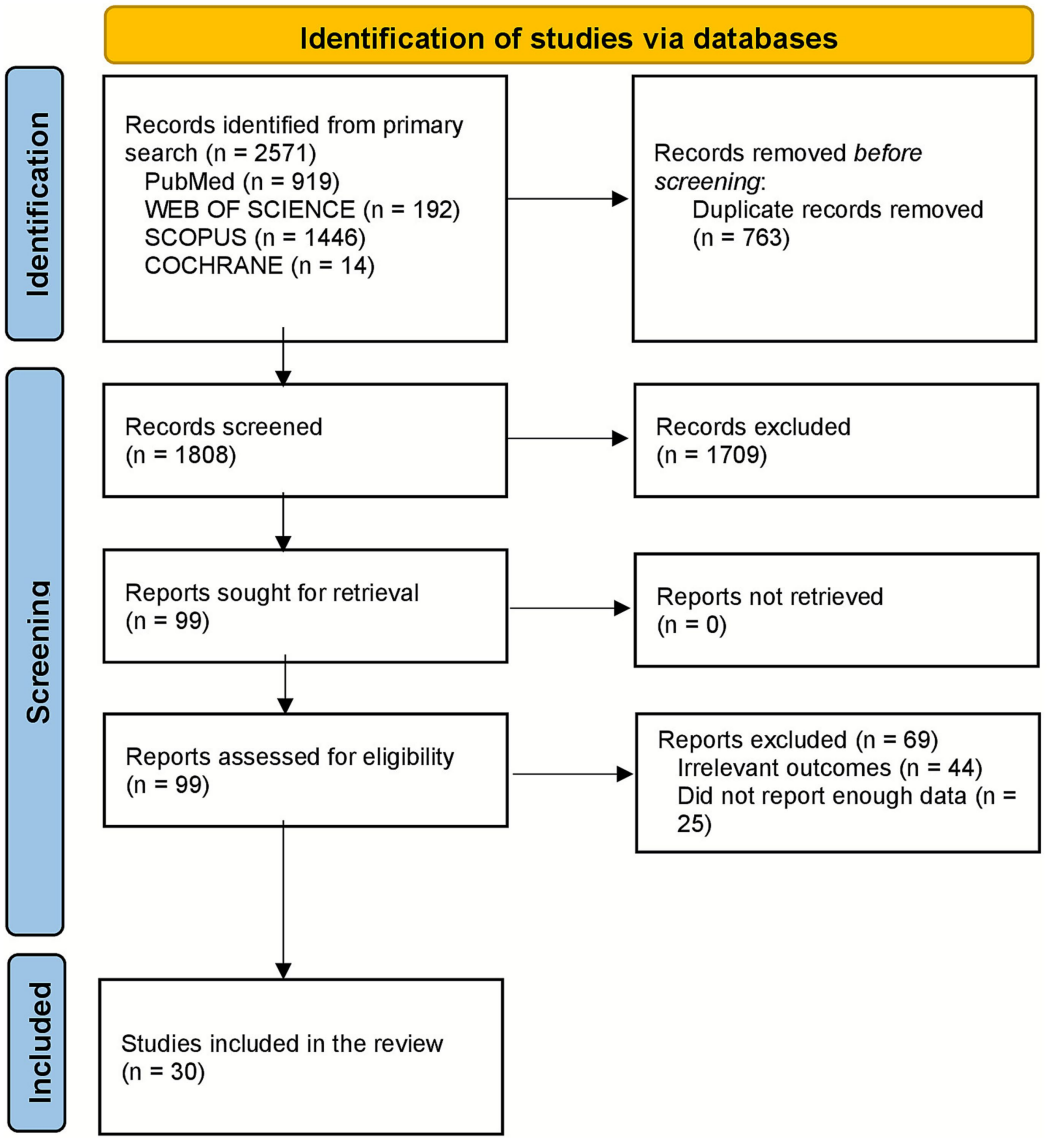
The PRISMA flow diagram for database search and study selection.

The systematic review commenced with an initial search across four databases (PubMed, Web of Science, Scopus, and Cochrane), identifying 2,571 records. After removing 763 duplicates, 1,808 records remained for screening. Of these, 1,709 were excluded based on title and abstract review, leaving 99 articles for full-text assessment. Following a detailed evaluation, 59 studies were excluded due to irrelevant outcomes (*n* = 44) or insufficient data reporting (*n* = 25). In total, 30 studies met the eligibility criteria and were included in the systematic review and meta-analysis (9, 13, 14, 17, 23–48). This methodical selection process, illustrated in the PRISMA flow diagram (Figure 1), ensured the inclusion of relevant, high-quality studies for analysis.

### Main characteristics of the included studies

This systematic review included 30 studies published between 2010 and 2024, spanning diverse geographic regions, with a predominance of studies from China (*n* = 11), Nigeria (*n* = 4), and Pakistan (*n* = 4). Sample sizes varied widely, ranging from 58 to 69,674 participants. The majority of studies (*n* = 18) used a retrospective design, while 10 were prospective and 5 were cross-sectional. Hyperuricemia cutoffs were primarily >6 mg/dL (*n* = 20) or >5 mg/dL (*n* = 8), with sampling predominantly in the third trimester (*n* = 18). Maternal age categories were frequently reported as 25–29 or 30–34 years. Quality assessment using the Newcastle–Ottawa Scale (NOS) revealed high-quality studies (scores 7–9; *n* = 14), moderate quality (scores 4–6; *n* = 15), and one low-quality study (score <4). Key outcomes focused on associations between maternal hyperuricemia and adverse fetal/maternal outcomes, with higher-quality studies demonstrating robust methodologies in selection and comparability adjustments (Table 1).

**Table 1:** Main characteristics of the included studies.

Study (ref)	Country	*n*	Study design	Hyperuricemia cutoff	Trimester at sampling	Maternal age	Newcastle–Ottawa Scale (NOS) Quality assessment			
							Selection	Comparability	Outcome	Score*^§^*
Adu-Bonsaffoh et al., 2024 (23)	Ghana	100	Cross-sectional	>6 mg/dL	Third	25–29	***	**	**	7
Ahmed et al., 2024 (24)	Egypt	221	Prospective	>5 mg/dl	Third	30–34	***	**	*	6
Akgün et al., 2024 (25)	Turkey	394	Retrospective	>5 mg/dl	Third	27–31	**	**	**	6
Allagoa et al., 2020 (26)	Nigeria	200	Prospective	>6 mg/dl	Third	28–31	**	**	**	6
Ayankunle et al., 2022 (27)	Nigeria	110	Prospective	>6 mg/dL	Third	30–31	****	**	***	9
Bai et al., 2020 (28)	Pakistan	237	Prospective	>6 mg/dl	Second	25–29	**	**	*	5
Chen et al., 2010 (29)	Taiwan	19,566	Retrospective	>6 mg/dl	First	30–34	**	*	*	4
de Mendonça et al., 2022 (30)	Brazil	267	Cross-sectional	>6 mg/dl	First, second, and third	25–29	***	**	**	7
Gajani et al., 2022 (31)	Pakistan	170	Cross-sectional	>5 mg/dl	Second and third	31–32	**	**	**	6
Germany Paula et al., 2008 (32)	Brazil	58	Retrospective	>6 mg/dl	Third	25–29	**	**	*	5
Hawkins et al., 2012 (13)	Australia	1880	Retrospective	>5 mg/dl	First, second, and third	-	**	**	**	6
Laughon et al., 2011 (33)	United States	1,541	Prospective	>6 mg/dl	First	25–29	***	**	**	7
Lawal et al., 2020 (34)	Nigeria	100	Prospective	>6 mg/dl	Third	26–27	**	**	**	6
Le et al., 2018 (17)	Vietnam	205	Retrospective	>5 mg/dl	Second	30.6 ± 6.7	**	**	**	6
Lin et al., 2018 (35)	China	360	Prospective	>6 mg/dl	Third	30–34	***	**	***	8
Liu et al., 2019 (36)	China	6,715	Cross-sectional	>6 mg/dl	Third	25–29	****	**	**	8
Luo et al., 2024 (14)	China	692	Retrospective	>7 mg/dL	Third	35–39	**	**	**	6
Nadeem et al., 2022 (37)	Pakistan	60	Prospective	>5 mg/dl	First, second, and third	35–39	**	**	*	5
Ngeri et al., 2022 (38)	Nigeria	190	Prospective	>6 mg/dl	Third	25–29	***	**	**	7
Pang et al., 2023 (39)	China	18,250	Retrospective	>6 mg/dl	Second	25–29	****	**	***	9
Schmella et al., 2015 (40)	United States	259	Retrospective	>5 mg/dl	Third	20–24	**	**	***	7
Sudjai et al., 2022 (41)	Thailand	400	Retrospective	>7 mg/dl	First, second, and third	30–34	**	**	**	6
Ugwuanyi et al., 2021 (42)	Nigeria	102	Retrospective	>6 mg/dl	Second	25–29	***	**	**	7
Wu et al., 2022 (43)	China	1,602	Retrospective	>6 mg/dl	Third	30–34	***	**	***	8
Xiong et al., 2024 (44)	China	69,674	Prospective	>6 mg/dl	First, second, and third	30–34	****	**	**	8
Yan et al., 2024 (45)	China	1,057	Retrospective	>6 mg/dl	First, second, and third	30–34	***	**	**	7
Yang et al., 2023 (46)	China	1,010	Prospective	>6 mg/dl	Third	25–29	**	**	*	5
Yuan et al., 2022 (9)	China	11,580	Retrospective	>6 mg/dl	Third	25–29	****	**	*	7
Yue et al., 2023 (47)	China	4,725	Retrospective	>5 mg/dl	First	25–29	***	**	***	8
Zaineb et al., 2023 (48)	Pakistan	106	Cross-sectional	>6 mg/dL	Third	25–26	**	**	**	6

^§^ NOS scoring; maximum score: 9 points. Studies scoring 7–9 points are generally considered high quality. Studies scoring 4–6 points are considered moderate quality. Studies scoring less than 4 points are considered low quality.

### Meta-analysis

**Figure 2:**
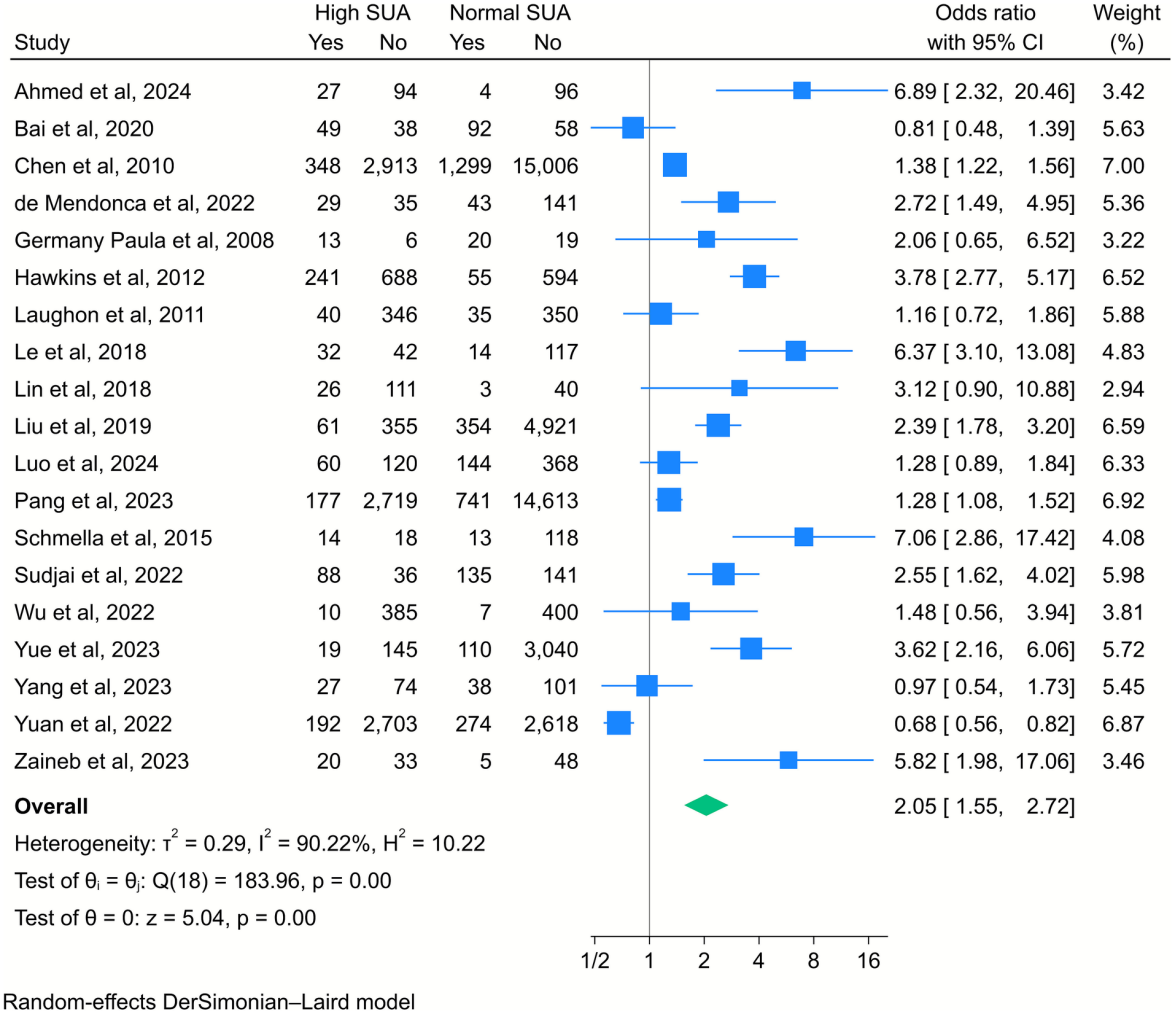
Forest plot of the correlation between high serum uric acid (SUA) and preterm birth. Data are presented as odds ratios with 95% confidence intervals (CI).

A total of 19 studies included in the analysis evaluated the correlation between high SUA levels and the risk of PTB. The meta-analysis revealed that elevated SUA levels were significantly associated with increased odds of PTB (OR = 2.05, 95% CI [1.55, 2.72], *k* = 19, *I*^2^ = 90.22%, *τ*^2^ = 0.29; Figure 2). The subgroup analysis further showed that this association was particularly significant among pregnant women in the third trimester compared to those in the first or second trimesters (Supplementary File 2).

**Figure 3:**
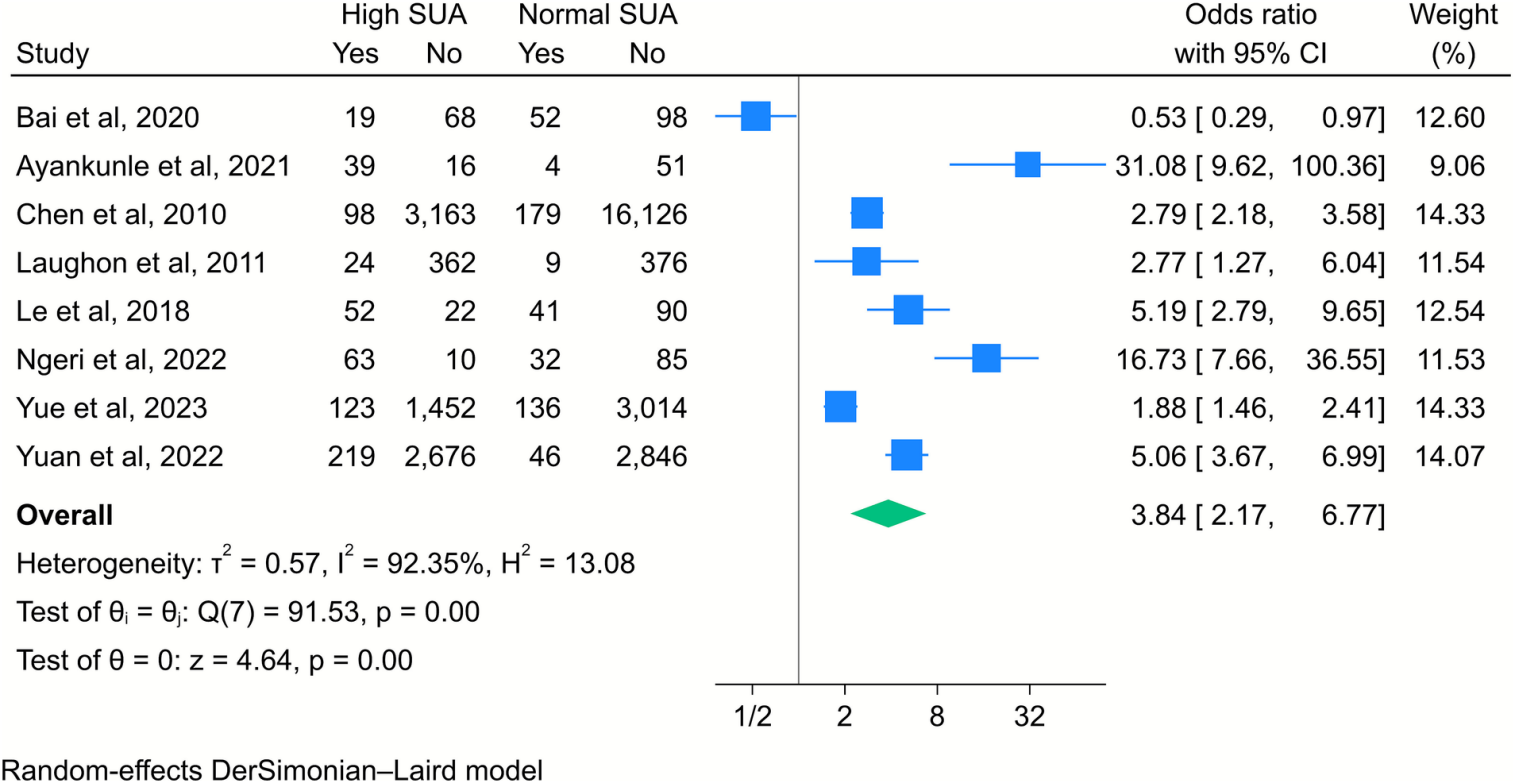
Forest plot of the correlation between high serum uric acid (SUA) and preeclampsia. Data are presented as odds ratios with 95% confidence intervals (CI).

The pooled results of eight studies also indicated that high SUA levels were significantly associated with an increased risk of PE (OR = 3.8, 95% CI [2.17, 6.77], *k* = 8, *I*^2^ = 92.35%, *τ*^2^ = 0.57; Figure 3). The subgroup analysis revealed that this association was particularly evident among women in the first and third trimesters of pregnancy (Supplementary File 3).

**Figure 4:**
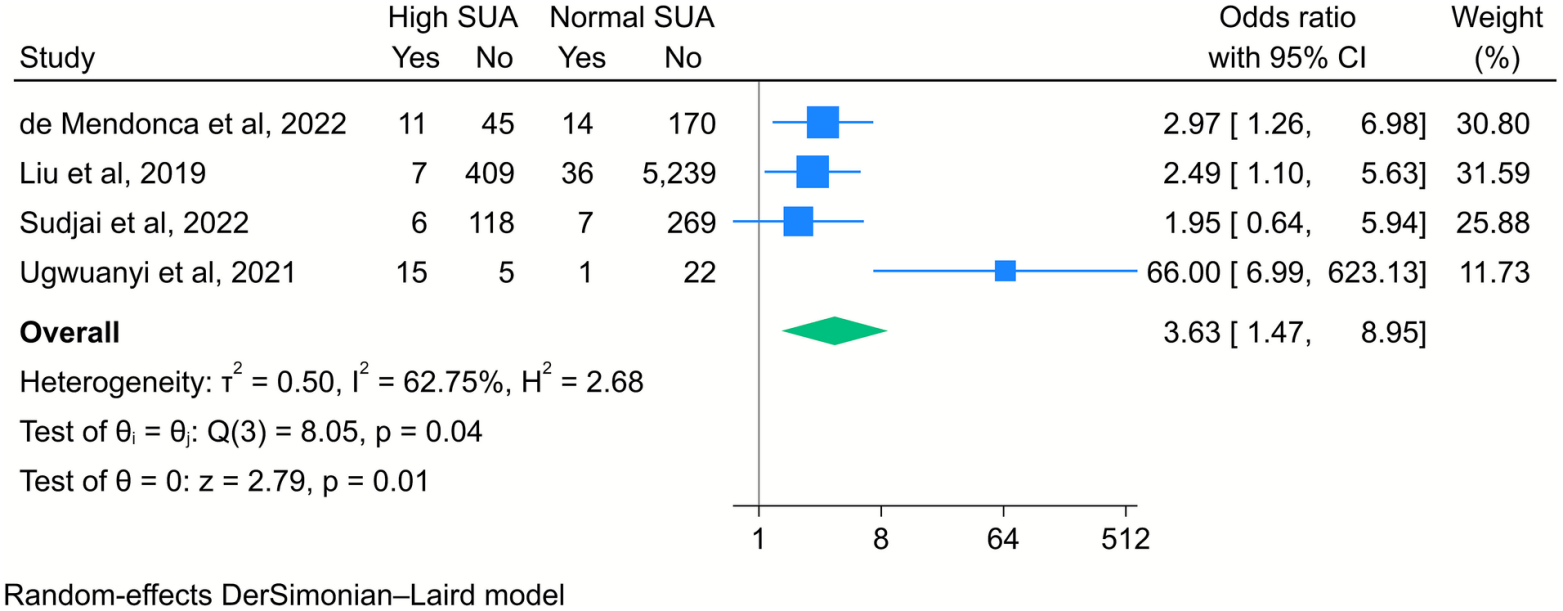
Forest plot of the correlation between high serum uric acid (SUA) and the APGAR score at 1 min. Data are presented as odds ratios with 95% confidence intervals (CI).

**Figure 5:**
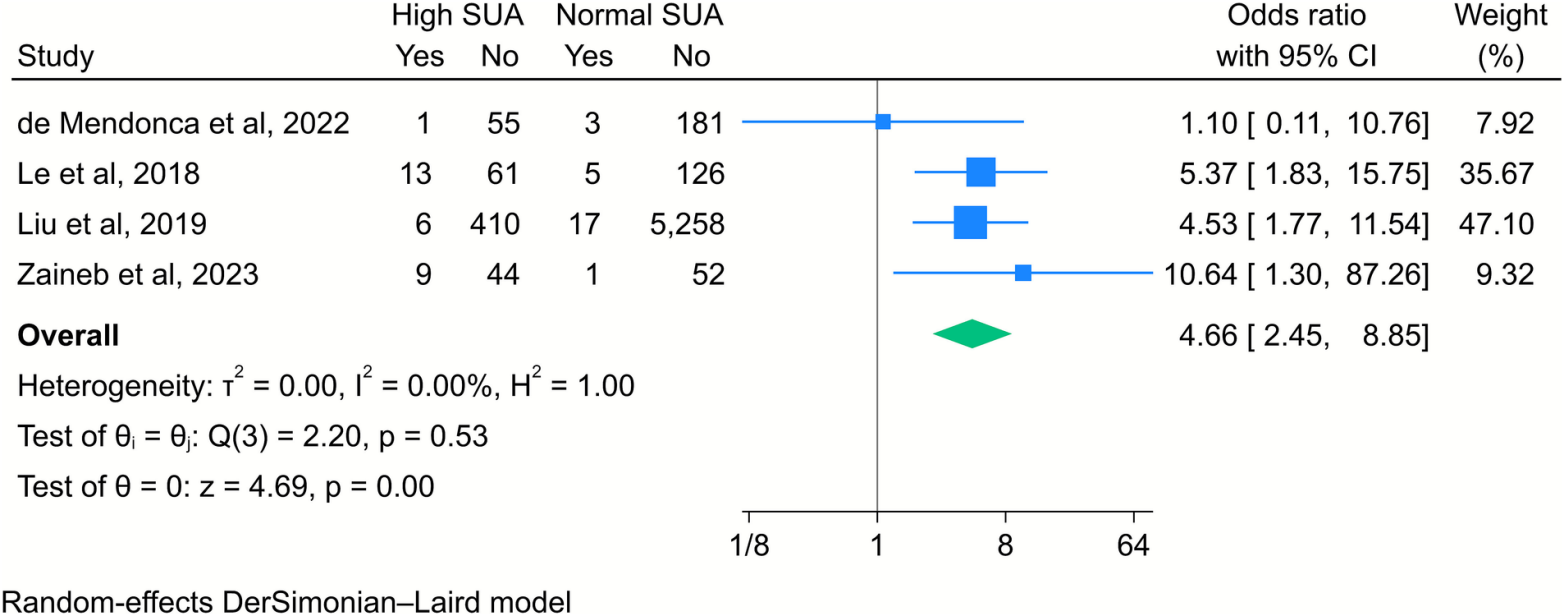
Forest plot of the correlation between high serum uric acid (SUA) and the APGAR score at 5 min. Data are presented as odds ratios with 95% confidence intervals (CI).

Four included studies revealed that elevated maternal SUA levels significantly increased the risk of low APGAR scores in newborns. Specifically, the risk was higher at both 1 min (OR = 3.6, 95% CI [1.47, 8.95], *k* = 4, *I*^2^ = 62.75%, *τ*^2^ = 0.50; Figure 4) and 5 min after birth (OR = 4.66, 95% CI [2.45, 8.85], *k* = 4, *I*^2^ = 0%, *τ*^2^ = 0; Figure 5).

**Figure 6:**
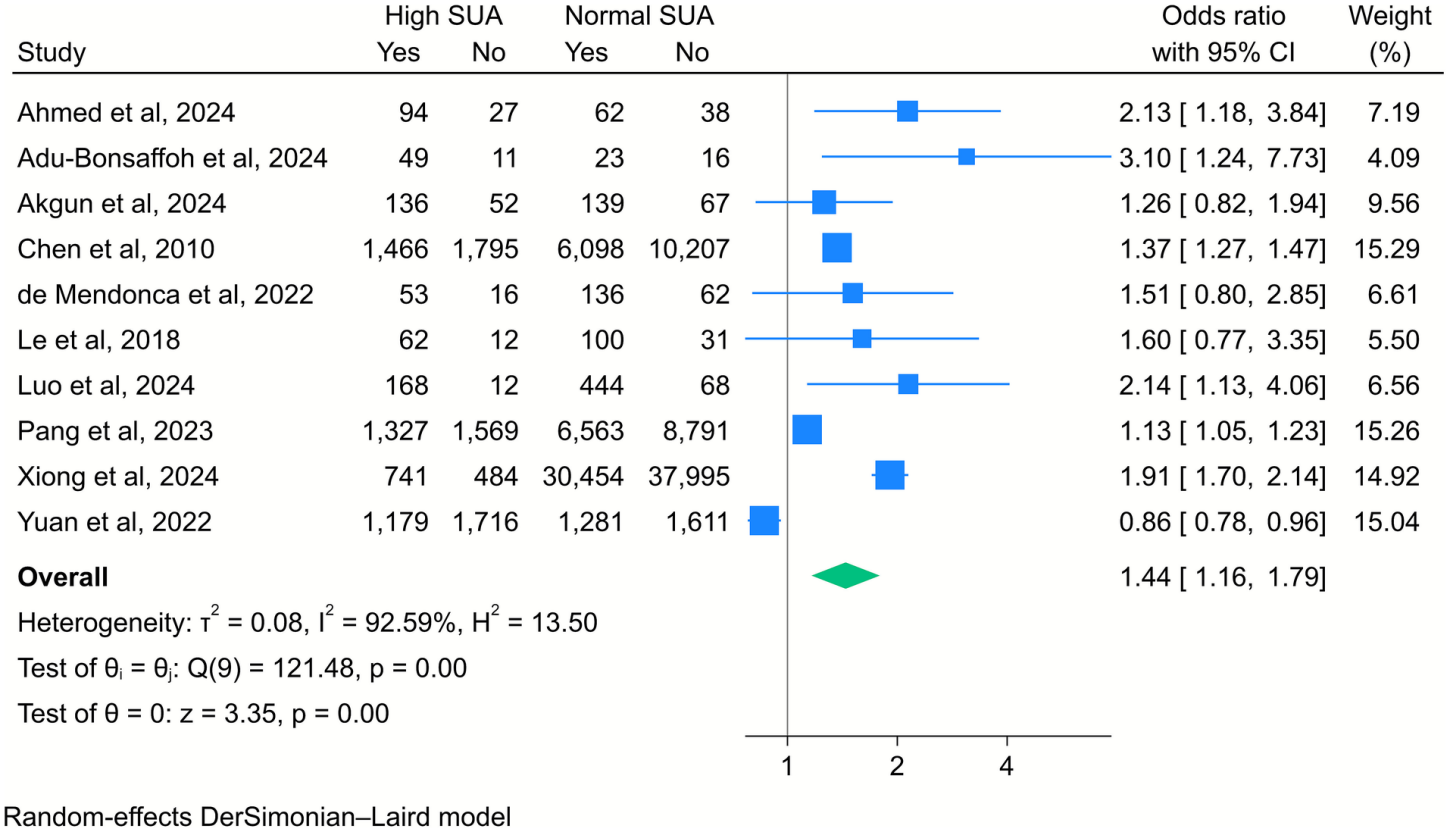
Forest plot of the correlation between high serum uric acid (SUA) and cesarean section. Data are presented as odds ratios with 95% confidence intervals (CI).

Additionally, a meta-analysis of 10 studies showed a significant association between high SUA levels and increased risk of cesarean delivery (OR = 1.44, 95% CI [1.16, 1.79], *k* = 10, *I*^2^ = 92.59%, *τ*^2^ = 0.08; Figure 6). The subgroup analysis indicated that this association remained significant, particularly among middle-aged mothers compared to younger ones (Supplementary File 4).

**Figure 7:**
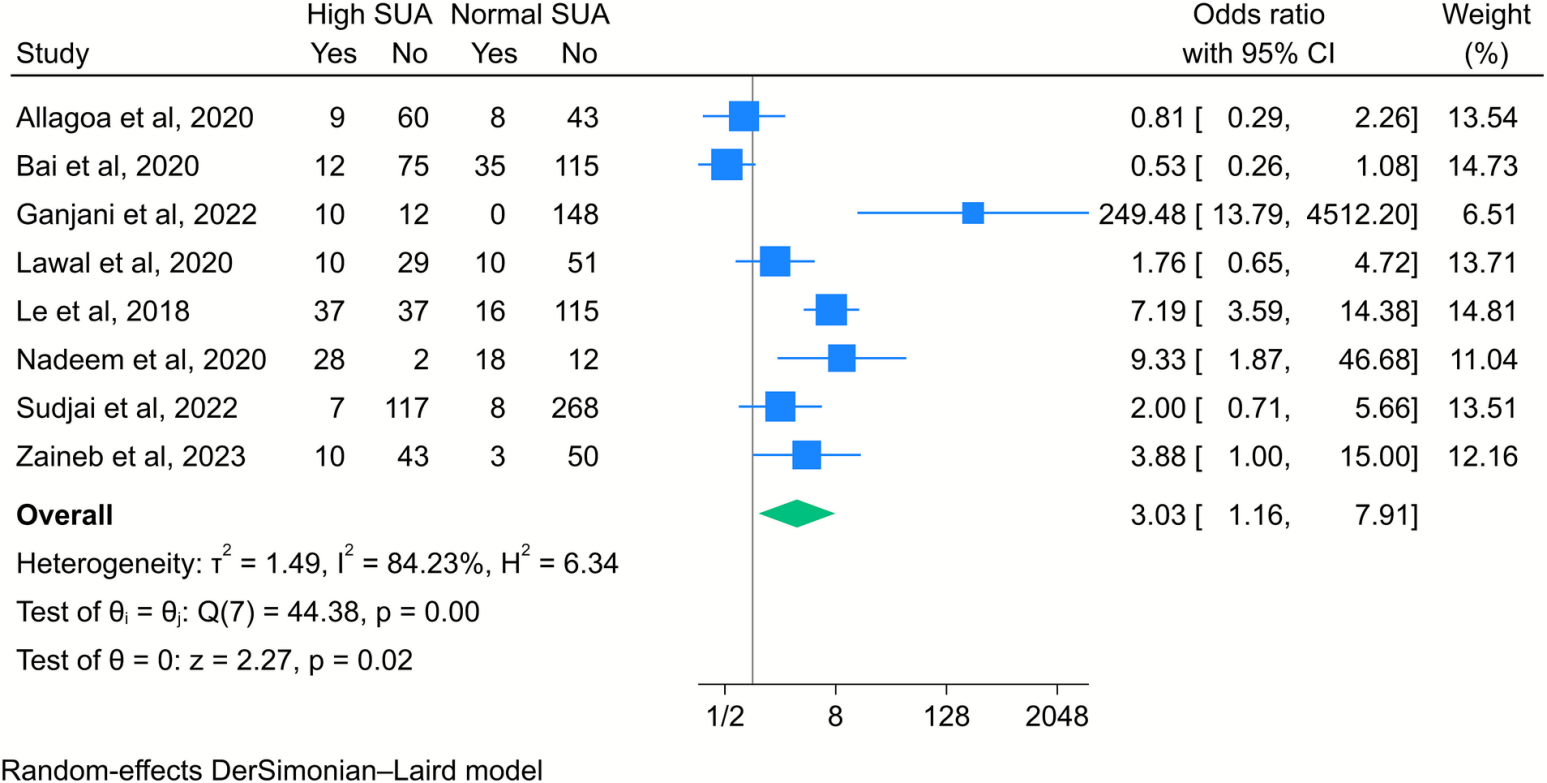
Forest plot of the correlation between high serum uric acid (SUA) and intrauterine growth restriction. Data are presented as odds ratios with 95% confidence intervals (CI).

The results of another eight studies showed a significantly increased risk of IUGR among mothers with elevated SUA levels (OR = 3.03, 95% CI [1.16, 7.91], *k* = 8, *I*^2^ = 84.23%, *τ*^2^ = 1.49; Figure 7). The subgroup analysis showed that this association was stronger when a high SUA level was defined as greater than 5 mg/dL, compared to higher thresholds such as 6 mg/dL. The risk was also higher among middle-aged mothers than younger mothers (Supplementary File 5).

**Figure 8:**
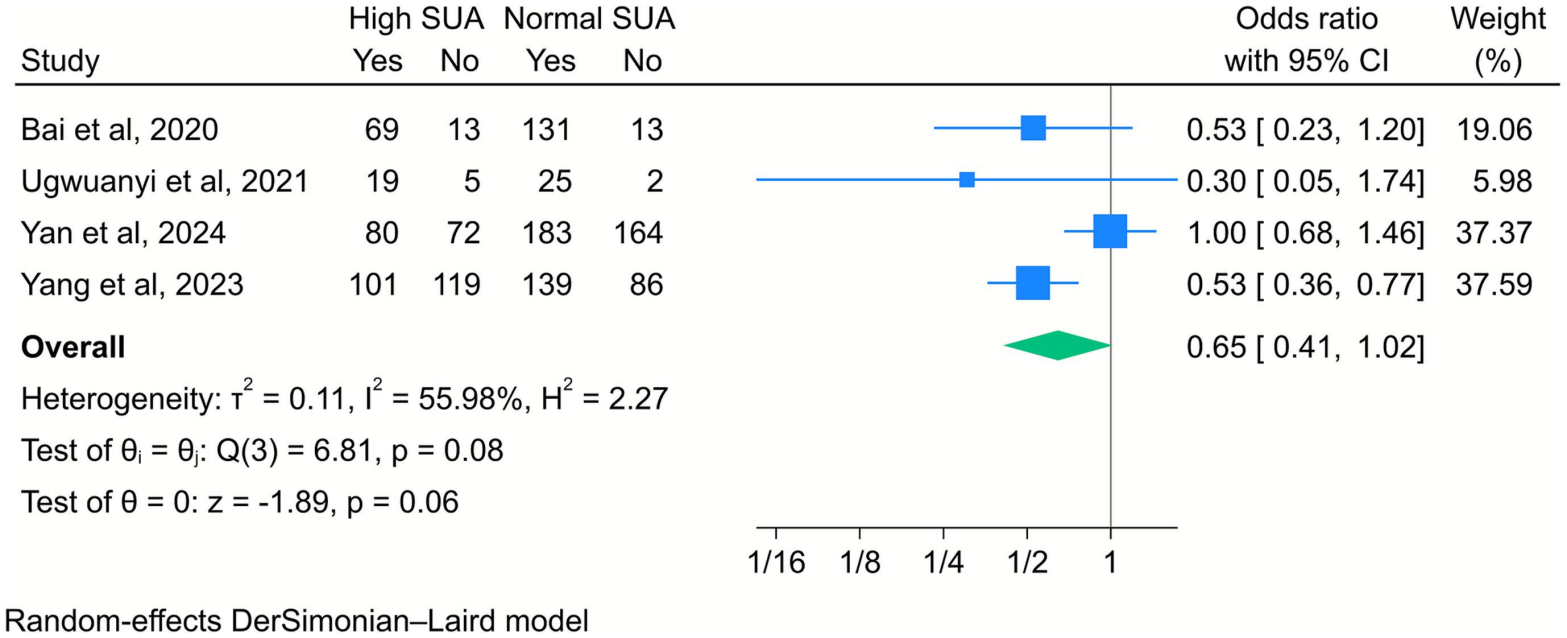
Forest plot of the correlation between high serum uric acid (SUA) and live birth. Data are presented as odds ratios with 95% confidence intervals (CI).

In contrast, four included studies showed that there was no statistically significant association between high maternal SUA levels and live birth; however, a downward trend was observed (OR = 0.65, 95% CI [0.41, 1.02], *k* = 4, *I*^2^ = 55.98%, *τ*^2^ = 0.11; Figure 8).

**Figure 9:**
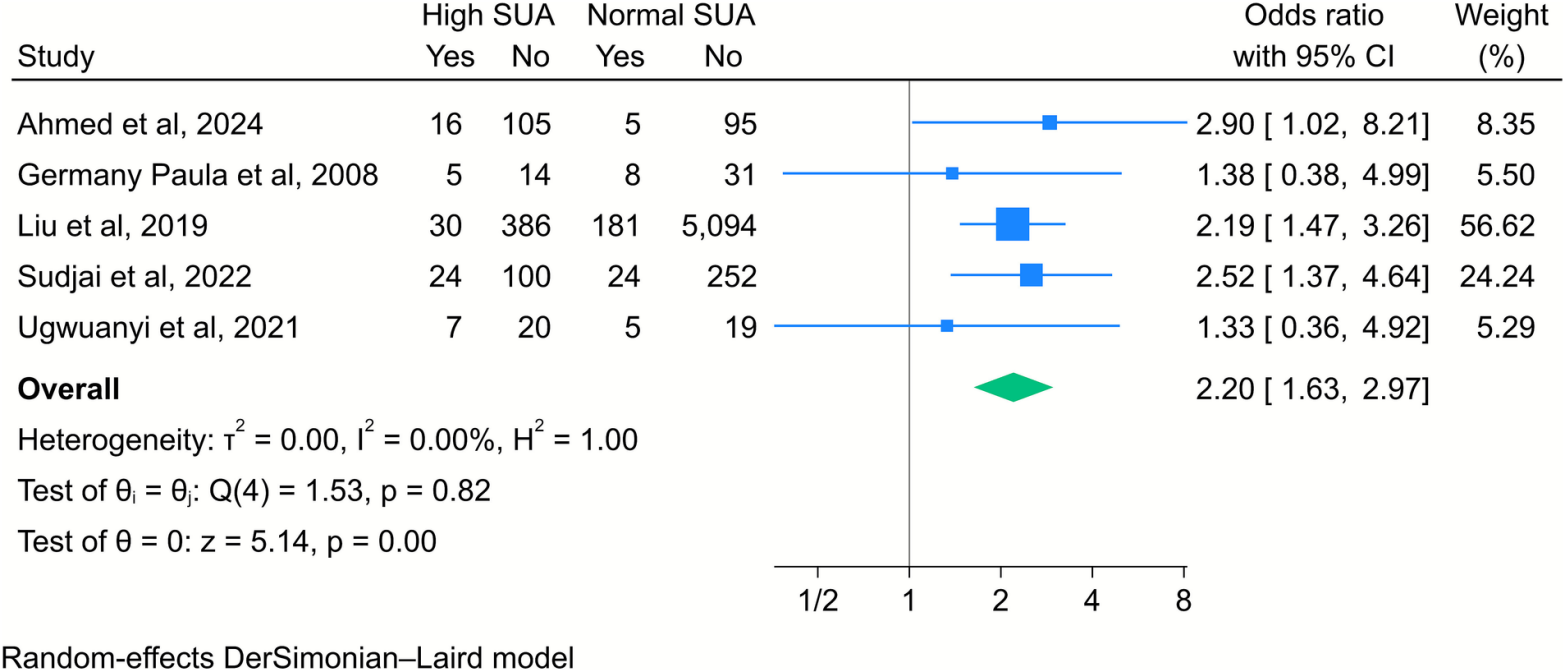
Forest plot of the correlation between high serum uric acid (SUA) and neonatal intensive care unit admission. Data are presented as odds ratios with 95% confidence intervals (CI).

Furthermore, high maternal SUA levels were significantly associated with an increased risk of NICU admission (OR = 2.2, 95% CI [1.63, 2.97], *k* = 5, *I*^2^ = 0%, *τ*^2^ = 0; Figure 9).

**Figure 10:**
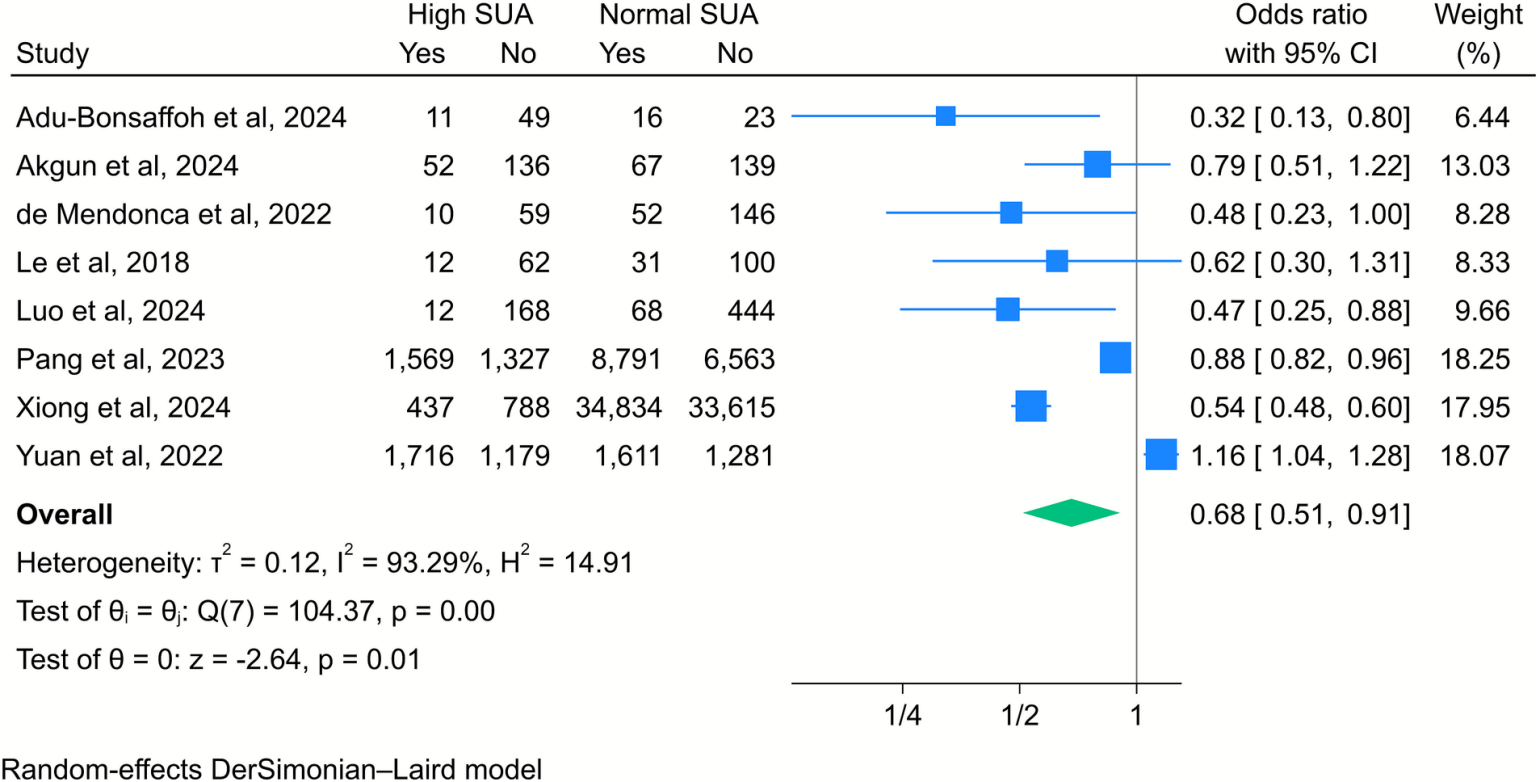
Forest plot of the correlation between high serum uric acid (SUA) and spontaneous vaginal delivery. Data are presented as odds ratios with 95% confidence intervals (CI).

Finally, elevated SUA levels were correlated with a reduced likelihood of SVD (OR = 0.68, 95% CI [0.51, 0.91], *k* = 8, *I*^2^ = 93.29%, *τ*^2^ = 0.12; Figure 10). The subgroup analysis showed that this negative association remained significant among mothers whose SUA levels were measured in the second trimester, as compared to those in the third trimester. The association was also stronger in studies that defined high SUA levels as greater than 6 mg/dL and among middle-aged mothers compared to younger ones (Supplementary File 6).

### Publication bias, sensitivity analysis, and certainty of evidence

To assess the effect of each included study on the pooled effect size of our outcomes of interest, we performed a sensitivity analysis, and the results showed that there were no significant changes in the overall effect size after removing each one of the studies for all variables of interest (Supplementary File 7). We also performed a meta-regression analysis to identify potential sources of heterogeneity in our variables, the results of which are presented in Supplementary File 8. Visual inspection of funnel plots and formal statistical testing were used to assess publication bias only for outcomes with ≥10 included studies. For PTB, Begg’s test did not indicate significant publication bias (*p* = 0.269), whereas Egger’s test suggested evidence of small-study effects (*p* = 0.018). For cesarean section, neither Begg’s test (*p* = 0.5915) nor Egger’s test (*p* = 0.0622) indicated significant publication bias (Supplementary File 8).

The GRADE assessment of our meta-analysis reported outcome-specific certainty ratings with explicit, domain-level downgrading decisions (Supplementary File 9). For PTB (19 studies) and PE (8 studies), the certainty of evidence was rated as moderate, with downgrading by one level for inconsistency due to substantial heterogeneity (*I*^2^ > 60%), while no downgrading was applied for risk of bias, indirectness, imprecision, or publication bias. Outcomes with fewer studies showed lower certainty. The APGAR score at 1 min (4 studies) was rated as very low certainty, downgraded for inconsistency (high heterogeneity), indirectness (limited number of studies and variability in outcome assessment), and imprecision (wide confidence intervals). The APGAR score at 5 min (4 studies) was rated as low certainty, downgraded for indirectness and imprecision. Cesarean section (10 studies) and natural vaginal delivery (8 studies) were rated as moderate certainty, downgraded only for inconsistency. NICU admission (5 studies) and IUGR (8 studies) were rated as low certainty, downgraded for indirectness (limited number of contributing studies) and imprecision (wide confidence intervals). Live birth (four studies) was rated as very low certainty, downgraded for inconsistency, indirectness, and imprecision, including lack of statistical significance. Risk of bias information within the GRADE was provided by the NOS, with NOS domains mapped transparently to GRADE criteria (selection → risk of bias; comparability → control of confounding; and outcome/exposure assessment → measurement bias). Since the majority of studies achieved moderate-to-high NOS scores, no outcome was downgraded for risk of bias. Publication bias was not downgraded for any outcome, as formal testing was conducted only when ≥10 studies were available and did not consistently indicate bias.

## Discussion

This systematic review and meta-analysis synthesizes evidence from 30 studies evaluating the correlation between maternal SUA levels and a range of adverse pregnancy and neonatal outcomes. Our findings indicate that elevated maternal SUA levels are significantly associated with increased risks of PTB, PE, IUGR, low APGAR scores, cesarean section, NICU admission, and reduced chance of SVD. Although the association with live birth was not statistically significant, a downward trend was observed. These results highlight the potential of maternal SUA as a valuable biomarker for identifying women at higher risk for obstetric and neonatal complications. Given the simplicity and cost-effectiveness of SUA testing, our findings highlight the clinical relevance of monitoring uric acid levels during pregnancy as part of routine maternal care, particularly in middle to late gestation.

Our findings are largely in line with prior studies that have reported a link between hyperuricemia and adverse pregnancy outcomes (4, 49). Consistent with our results, previous meta-analyses have shown a robust association between elevated SUA levels and the risk of PE and PTB. However, there are notable differences in the strength of these associations and in the range of outcomes evaluated. For instance, while some earlier reviews focused solely on PE or PTB, our study provides a more comprehensive assessment by including additional outcomes such as IUGR, APGAR scores, and SVD. The stronger associations we observed in the third trimester and among middle-aged mothers may help explain discrepancies with earlier literature. Differences in study design, SUA cutoff values, geographic regions, and confounder adjustments likely contributed to the heterogeneity of findings. Moreover, by incorporating a larger, more recent, and methodologically rigorous body of evidence, our systematic review offers an updated and more refined perspective on these associations.

Our results regarding the significant correlation between elevated maternal SUA levels and an increased risk of PE and adverse pregnancy outcomes are consistent with several previous studies. Pecoraro et al. (16), Colmenares-Mejia et al. (50), Bellos et al. (51), and Piani et al. (2023) (52) reported significant associations between elevated uric acid levels and PE, supporting the robustness of this relationship across different gestational periods and population subgroups. Notably, Colmenares-Mejia et al. (50) further confirmed a positive linear relationship using early pregnancy uric acid levels (<20 weeks’ gestation), which aligns with our subgroup analysis. Similarly, Bellos et al. (51) emphasized the predictive value of uric acid across different trimesters in identifying pregnancy complications, while Pecoraro et al. (16) acknowledged the diagnostic potential of uric acid. However, our results extend beyond PE, supporting broader correlations with multiple maternal and neonatal complications. Additionally, our results align with those of Tan et al. (53) and Piani et al. (52), who also observed associations between hyperuricemia and adverse outcomes such as PTB and LBW. Although Tan et al. (53) did not find a significant relationship with SGA, our analysis showed a significant association with IUGR, which may reflect differences in outcome definitions or population characteristics.

High SUA levels may contribute to adverse pregnancy and neonatal outcomes through multiple interconnected mechanisms. Elevated SUA levels during pregnancy are increasingly recognized as a key contributor to a range of adverse maternal and neonatal outcomes, mediated by a complex interplay of interrelated biological mechanisms (54, 55). One of the primary effects of high SUA levels is the enhancing of systemic inflammation and oxidative stress, primarily driven by increased xanthine oxidoreductase (XOR) activity and excessive production of reactive oxygen species (ROS) (56, 57). This pro-oxidative environment disrupts endothelial function and impairs nitric oxide (NO) synthesis, leading to vascular constriction and compromised blood flow, particularly within the placenta (58–60). Simultaneously, SUA impairs insulin signaling pathways, contributing to insulin resistance and increasing the risk of gestational diabetes and PTB (61, 62). At the molecular level, uric acid activates signaling cascades such as NF-κB and ERK, upregulates the NLRP3 inflammasome, and induces mitochondrial dysfunction (63–65) and trophoblast apoptosis—processes that collectively impair spiral artery remodeling, a crucial step for adequate placental development and oxygen delivery to the fetus (66–68). In pregnant women with PE, high SUA levels are closely associated with placental dysfunction, reflected in elevated levels of anti-angiogenic factors such as sFlt-1 and reduced VEGF, leading to proteinuria, hypertension, and IUGR (69, 70). In addition, SUA’s ability to cross the placenta may exert direct effects on fetal development, heightening the risk of outcomes such as low birth weight, small for gestational age, and prematurity (71, 72). These physiological disruptions also appear to affect neonatal health more broadly, with evidence suggesting associations between high maternal SUA levels and low APGAR scores, increased rates of cesarean section, and greater need for NICU admission (14, 27). Taken together, these results highlight the multifaceted role of uric acid as both a marker and mediator of vascular, metabolic, and inflammatory disturbances that compromise pregnancy outcomes.

It has been demonstrated that abnormally low SUA levels are associated with adverse clinical outcomes, including increased short-term mortality and frequent obstetric presentations in emergency settings (73), and other evidence has reported that elevated SUA levels are associated with an increased risk of ischemic stroke, whereas low levels may be protective, underscoring the broader clinical relevance of uric acid dysregulation beyond pregnancy-specific outcomes (74). In this context, although our findings reveal statistically robust associations between elevated SUA levels and adverse pregnancy outcomes, the clinical role of SUA is best interpreted as a risk stratification marker rather than a standalone diagnostic test. Across the included studies, hyperuricemia was most commonly defined using thresholds of ≥5 mg/dL or ≥6 mg/dL, with stronger associations observed when the SUA level was measured in the third trimester, suggesting that gestational timing may influence the clinical relevance of SUA thresholds. However, these cutoffs should be viewed as indicators of risk enrichment rather than validated screening thresholds, as formal estimates of sensitivity and specificity could not be derived due to the lack of individual participant data and inconsistent outcome reporting across studies. Importantly, although elevated SUA levels are biologically associated with endothelial dysfunction, placental ischemia, and oxidative stress, evidence supporting its incremental predictive value beyond established clinical and biochemical markers such as blood pressure, proteinuria, or angiogenic biomarkers (e.g., sFlt-1/PlGF) remains limited. The majority of the included studies did not report multivariable prediction models incorporating these established predictors, precluding formal assessment of additive prognostic utility.

This systematic review and meta-analysis has several notable strengths. It is among the most comprehensive reviews to date, including 30 studies from diverse regions, using a rigorous search strategy across four major databases, and encompassing a wide range of variables and outcomes related to maternal and neonatal health. Our inclusion of multiple relevant outcomes and the conduct of subgroup analyses based on maternal age, gestational trimester, and SUA thresholds provide greater depth to our findings. Furthermore, sensitivity analyses and publication bias assessments strengthen the robustness of our conclusions.

However, our study also has limitations. First, the number of the included studies was limited for certain outcomes, including APGAR scores and NICU admission, which may reduce the reliability of these pooled estimates. Second, several pooled analyses showed extremely high between-study heterogeneity, with *I*^2^ values exceeding 90% for key outcomes such as PTB. This substantial heterogeneity limits the precision and interpretability of pooled effect estimates and suggests that true effects may vary considerably across populations and settings. Potential sources of heterogeneity include differences in study design, timing of SUA measurement, definitions and cutoff values for hyperuricemia, population characteristics, geographic regions, and unmeasured confounding. Although subgroup analyses and meta-regression were conducted to explore these sources, residual heterogeneity persisted, and the results should therefore be interpreted with appropriate caution. Third, the observational nature of the included studies prevents causal inference, and confounding factors may not have been fully accounted for in all analyses. Fourth, limited data availability in the included studies restricted our ability to perform additional subgroup analyses based on pre-pregnancy SUA levels and the socio-economic status of pregnant women. Finally, emerging evidence suggests that the serum uric acid-to-creatinine ratio (SUA/sCr) may better account for renal function and improve risk stratification compared with SUA alone and warrants evaluation in future pregnancy-focused studies.

## Conclusion

Our meta-analysis provides evidence of an association between elevated maternal SUA levels and several adverse maternal and neonatal outcomes, including PTB, PE, IUGR, low APGAR scores, increased risk of cesarean delivery, and NICU admission. However, these findings are the results of observational studies that exhibit substantial heterogeneity and should not be interpreted as evidence of causal effects or predictive clinical utility. At present, the available evidence is insufficient to support routine SUA-based risk stratification or screening in clinical practice. SUA may represent a promising biological marker associated with adverse pregnancy outcomes, but its role should be considered hypothesis-generating rather than practice-changing. Future research should aim to validate these associations in well-designed, large prospective cohort studies with standardized SUA measurements and comprehensive adjustment for confounding factors and to evaluate whether SUA adds incremental value beyond established clinical and biochemical predictors. Interventional and mechanistic studies are also required before any consideration of integrating SUA monitoring into prenatal care guidelines.

## Data Availability

The original contributions presented in the study are included in the article/Supplementary material, further inquiries can be directed to the corresponding author.
