# Supplementary material for: Maternal hyperuricemia and adverse maternal-fetal outcomes: a systematic review and meta-analysis of observational studies
Source: Front Med (Lausanne). 2026 Mar 9;13:1704136. doi: 10.3389/fmed.2026.1704136 (PMC13006587; doi:10.3389/fmed.2026.1704136)
Supplement: Supplementary file 1 [file Table_1.DOC]

**Supplementary File 1**. The specific literature search strategy.

| **Groups** | **Descriptors** |
| --- | --- |
| Outcome | “Pregnancy Outcome” OR “Premature Birth” OR “Preterm Birth” OR “Stillbirth” OR “Intrauterine Fetal Death” OR “IUFD” OR “Preeclampsia” OR “NICU admission” OR “Cesarean delivery” OR “Fetal Growth Retardation” OR “IUGR” OR “APGAR” OR “Normal vaginal delivery” OR “live birth” |
| Exposure | “Uric Acid” OR “Urate” OR “Trioxopurine” OR “Hyperuricemia” OR “Hyperuri*” OR “Hypouri*” |

**PUBMED**

**Number of localized studies:** 1074

**Limits:** humans

**Number of studies after applying limits:** 919

|  | **Descriptors** | Number of studies reached |
| --- | --- | --- |
| **#1** | "pregnancy outcome"[MeSH Terms] OR "pregnancy outcome"[All Fields] OR ("pregnancy"[All Fields] AND "outcome"[All Fields]) OR "premature birth"[MeSH Terms] OR "premature birth"[All Fields] OR "preterm birth"[All Fields] OR "preterm delivery"[All Fields] OR "stillbirth"[MeSH Terms] OR "stillbirth"[All Fields] OR "stillbirths"[All Fields] OR "intrauterine fetal death"[All Fields] OR "IUFD"[All Fields] OR ("intrauterine"[All Fields] AND "fetal"[All Fields] AND "death"[All Fields]) OR "fetal death"[MeSH Terms] OR "fetal death"[All Fields] OR "pre-eclampsia"[All Fields] OR "preeclampsia"[All Fields] OR "NICU admission"[All Fields] OR "NICU"[All Fields] OR "cesarean section"[MeSH Terms] OR "cesarean delivery"[All Fields] OR "c-section"[All Fields] OR "fetal growth retardation"[MeSH Terms] OR "IUGR"[All Fields] OR "fetal growth restriction"[All Fields] OR "growth restriction"[All Fields] OR "APGAR score"[MeSH Terms] OR "APGAR"[All Fields] OR ("low"[All Fields] AND "APGAR"[All Fields]) OR "normal vaginal delivery"[All Fields] OR "spontaneous vaginal delivery"[All Fields] OR "live birth"[MeSH Terms] OR "live birth"[All Fields] | 368577 |
| **#2** | "uric acid"[MeSH Terms] OR ("uric"[All Fields] AND "acid"[All Fields]) OR "uric acid"[All Fields] OR ("uratic"[All Fields] OR "uric acid"[MeSH Terms] OR ("uric"[All Fields] AND "acid"[All Fields]) OR "uric acid"[All Fields] OR "urate"[All Fields] OR "urates"[All Fields]) OR ("uric acid"[MeSH Terms] OR ("uric"[All Fields] AND "acid"[All Fields]) OR "uric acid"[All Fields] OR "trioxopurine"[All Fields]) OR ("hyperuricaemia"[All Fields] OR "hyperuricemia"[MeSH Terms] OR "hyperuricemia"[All Fields] OR "hyperuricemias"[All Fields]) OR "hyperuri*"[All Fields] OR "hypouri*"[All Fields] | 59135 |
| **#3** | **#1** AND **#2** | 1074 |

**WEB OF SCIENCE**

**Number of localized studies: 251**

**Limits:** documents types (articles)

**Number of studies after applying limits:** 192

|  | **Descriptors** | Number of studies reached |
| --- | --- | --- |
| **#1** | TS=("Pregnancy Outcome" OR "Premature Birth" OR "Preterm Birth" OR "Stillbirth" OR "Intrauterine Fetal Death" OR "IUFD" OR "Preeclampsia" OR "NICU admission" OR "Cesarean delivery" OR "Fetal Growth Retardation" OR "IUGR" OR "APGAR" OR "Normal vaginal delivery" OR "live birth") | 65211 |
| **#2** | TS=(“ Uric Acid”) OR TS=(“ Urate”) OR TS=(“ Trioxopurine”) OR TS=(“ Hyperuricemia”) OR TS=(“ Hyperuri*”) OR TS=(“ Hypouri*”) | 67268 |
| **#3** | **#1** AND **#2** | 251 |

**SCOPUS**

**Number of localized studies:** 1739

**Limits:** *document type* (article and article in press)

**Number of studies after applying limits:** 1446

|  | **Descriptors** | Number of studies reached |
| --- | --- | --- |
| **#1** | ( TITLE-ABS-KEY ( "Pregnancy Outcome" ) ) OR ( TITLE-ABS-KEY ( "Premature Birth" ) ) OR ( TITLE-ABS-KEY ( "Preterm Birth" ) ) OR ( TITLE-ABS-KEY ( "Stillbirth" ) ) OR ( TITLE-ABS-KEY ( "Intrauterine Fetal Death" ) ) OR ( TITLE-ABS-KEY ( iufd ) ) OR ( TITLE-ABS-KEY ( "Preeclampsia" ) ) OR ( TITLE-ABS-KEY ( "NICU admission" ) ) OR ( TITLE-ABS-KEY ( "Cesarean delivery" ) ) OR ( TITLE-ABS-KEY ( "Fetal Growth Retardation" ) ) OR ( TITLE-ABS-KEY ( iugr ) ) OR ( TITLE-ABS-KEY ( apgar ) ) OR ( TITLE-ABS-KEY ( "Normal vaginal delivery" ) ) OR ( TITLE-ABS-KEY ( "Live birth" ) ) | 310899 |
| **#2** | ( TITLE-ABS-KEY ( uric AND acid ) ) OR ( TITLE-ABS-KEY ( urate ) ) OR ( TITLE-ABS-KEY ( trioxopurine ) ) OR ( TITLE-ABS-KEY ( hyperuricemia ) ) OR ( TITLE-ABS-KEY ( hyperuri* ) ) OR ( TITLE-ABS-KEY ( hypouri* ) ) | 106101 |
| **#3** | **#1** AND **#2** | 1739 |

**COCHRANE**

**Number of localized studies: 14**

**Limits:** -

**Number of studies after applying limits:** 14

|  | **Descriptors** | Number of studies reached |
| --- | --- | --- |
| **#1** | ("Pregnancy Outcome"):ti,ab,kw OR ("Premature Birth"):ti,ab,kw OR ("Preterm Birth"):ti,ab,kw OR ("Stillbirth"):ti,ab,kw OR ("Intrauterine Fetal Death"):ti,ab,kw OR ("IUFD"):ti,ab,kw OR ("Preeclampsia"):ti,ab,kw OR ("NICU admission"):ti,ab,kw OR ("Cesarean delivery"):ti,ab,kw OR ("Fetal Growth Retardation"):ti,ab,kw OR ("IUGR"):ti,ab,kw OR ("APGAR"):ti,ab,kw OR ("Normal vaginal delivery"):ti,ab,kw OR ("Live Birth"):ti,ab,kw | 28107 |
| **#2** | Me ("Uric Acid") or ("Urate"):ti,ab,kw or ("Trioxopurine"):ti,ab,kw or ("Hyperuricemia"):ti,ab,kw or ("Hyperuri"):ti,ab,kw or ("Hypouri"):ti,ab,kw | 2459 |
| **#3** | **#1** AND **#2** | 14 |
