# Supplementary material for: Maternal hyperuricemia and adverse maternal-fetal outcomes: a systematic review and meta-analysis of observational studies
Source: Front Med (Lausanne). 2026 Mar 9;13:1704136. doi: 10.3389/fmed.2026.1704136 (PMC13006587; doi:10.3389/fmed.2026.1704136)
Supplement: Supplementary file 2 [file Table_2.DOCX]

**Supplementary File 2**. Forest plot of the correlation between high serum uric acid (SUA) with preterm birth (PTB) subgroups based on: **[A]** sampling trimester, **[B]** cut-off level of SUA (mg/dl), and **[C]** maternal age (years).

**[A]**

**[B]**

**[C]**
