# Supplementary material for: Maternal hyperuricemia and adverse maternal-fetal outcomes: a systematic review and meta-analysis of observational studies
Source: Front Med (Lausanne). 2026 Mar 9;13:1704136. doi: 10.3389/fmed.2026.1704136 (PMC13006587; doi:10.3389/fmed.2026.1704136)
Supplement: Supplementary file 7 [file Table_7.DOCX]

**Supplementary File 7**. Sensitivity analysis of the correlation between high serum uric acid and: **[A]** preterm birth **[B]** preeclampsia, **[C]** APGAR score at 1 minutes, **[D]** APGAR score at 5 minutes, **[E]** cesarean section, **[F]** intrauterine growth restriction, **[G]** live birth, **[H]** neonatal intensive care unit admission, and **[I]** natural vaginal delivery. Data are presented as odds ratio with 95% confidence intervals (CI).

**[A]**

**[B]**

**[C]**

**[D]**

**[E]**

**[F]**

**[G]**

**[H]**

**[I]**
