# Supplementary material for: Maternal hyperuricemia and adverse maternal-fetal outcomes: a systematic review and meta-analysis of observational studies
Source: Front Med (Lausanne). 2026 Mar 9;13:1704136. doi: 10.3389/fmed.2026.1704136 (PMC13006587; doi:10.3389/fmed.2026.1704136)
Supplement: Supplementary file 8 [file Table_8.DOCX]

**Supplementary File 8**. Publication bias and meta-regression results.

**Supplementary Table S1**. Publication bias based on Begg’s and Egger’s tests.

| **Publication Bias (p values)** | **Begg’s test** | **Egger’s test** |
| --- | --- | --- |
| Preterm birth | 0.269 | **0.018** |
| Cesarean section | 0.5915 | 0.0622 |

**Supplementary Table S2.** Meta-regression based on sampling trimester, cut-off level (mg/dl) of serum uric acid (SUA), and maternal age in years.

| Outcomes | Regression analysis | | | | | |
| --- | --- | --- | --- | --- | --- | --- |
|  | SUA cut-off level | | Sampling trimester | | Maternal age | |
|  | Coefficient | p value | Coefficient | p value | Coefficient | p value |
| Preterm birth | **-0.408** | **0.007** | 0.202 | 0.516 | 0.195 | 0.237 |
| Preeclampsia | 0.052 | 0.782 | 0.461 | 0.123 | 0.133 | 0.494 |
| Cesarean section | -0.152 | 0.747 | 0.422 | 0.663 | 0.599 | 0.213 |
| Intrauterine growth restriction | -0.338 | 0.177 | 0.242 | 0.527 | 0.308 | 0.213 |
| Natural vaginal delivery | -0.126 | 0.792 | -0.705 | 0.363 | -0.618 | 0.240 |

**Supplementary Figure S1.** Funnel plot for publication bias: **[A]** preterm birth, **[B]** preeclampsia, **[C]** cesarean section, **[D]** intrauterine growth restriction, and **[E]** natural vaginal delivery.

**[A]**

**[B]**

**[C]**

**[D]**

**[E]**

**Supplementary Figure S2**. Meta regression of preterm birth based on: **[A]** cut-off level of serum uric acid (mg/dl), **[B]** sampling trimester, and **[C]** maternal age (year).

**[A]**

**[B]**

**[C]**

**Supplementary Figure S3**. Meta regression of preeclampsia based on: **[A]** cut-off level of serum uric acid (mg/dl), **[B]** sampling trimester, and **[C]** maternal age (year).

**[A]**

**[B]**

**[C]**

**Supplementary Figure S4**. Meta regression of cesarean section based on: **[A]** cut-off level of serum uric acid (mg/dl), **[B]** sampling trimester, and **[C]** maternal age (year).

**[A]**

**[B]**

**[C]**

**Supplementary Figure S5.** Meta regression of intrauterine growth restriction, based on: **[A]** cut-off level of serum uric acid (mg/dl), **[B]** sampling trimester, and **[C]** maternal age (year).

**[A]**

**[B]**

**[C]**

**Supplementary Figure S6**. Meta regression of natural vaginal delivery, based on: **[A]** cut-off level of serum uric acid (mg/dl), **[B]** sampling trimester, and **[C]** maternal age (year).

**[A]**

**[B]**

**[C]**
