# Supplementary material for: Maternal hyperuricemia and adverse maternal-fetal outcomes: a systematic review and meta-analysis of observational studies
Source: Front Med (Lausanne). 2026 Mar 9;13:1704136. doi: 10.3389/fmed.2026.1704136 (PMC13006587; doi:10.3389/fmed.2026.1704136)
Supplement: Supplementary file 9 [file Table_9.DOCX]

**Supplementary File 9**. GRADE approach to evaluate the certainty of evidence.

| **Certainty assessment** | | | | | | | **№ of patients** | | **Effect** | | **Certainty** | **Importance** |
| --- | --- | --- | --- | --- | --- | --- | --- | --- | --- | --- | --- | --- |
| **№ of studies** | **Study design** | **Risk of bias** | **Inconsistency** | **Indirectness** | **Imprecision** | **Other considerations** | **High SUA** | **Normal SUA** | **Relative (95% CI)** | **Absolute (95% CI)** |  |  |
| **Preterm Birth** | | | | | | | | | | | | |
| 19 | Non-RCTs | not serious | serious^a^ | not serious | not serious | none | 1453/13263 (11.0%) | 3391/45629 (7.4%) | **OR 2.05** (1.55 to 2.72) | **0 fewer per --** (from 0 fewer to 0 fewer) | ⨁⨁⨁◯ Moderate^a^ | CRITICAL |
| **Preeclampsia** | | | | | | | | | | | | |
| 8 | Non-RCTs | not serious | serious^a^ | not serious | not serious | none | 637/8406 (7.6%) | 499/23185 (2.2%) | **OR 3.84** (2.17 to 6.77) | **56 more per 1,000** (from 24 more to 108 more) | ⨁⨁⨁◯ Moderate^a^ | CRITICAL |
| **APGAR score at 1 minute** | | | | | | | | | | | | |
| 4 | Non-RCTs | not serious | serious^a^ | serious^b^ | serious^c^ | none | 39/616 (6.3%) | 58/5758 (1.0%) | **OR 3.63** (1.47 to 8.95) | **26 more per 1,000** (from 5 more to 73 more) | ⨁◯◯◯ Very low^a,b,c^ | CRITICAL |
| **APGAR score at 5 minutes** | | | | | | | | | | | | |
| 4 | Non-RCTs | not serious | not serious | serious^b^ | serious^c^ | none | 29/599 (4.8%) | 26/5643 (0.5%) | **OR 4.66** (2.45 to 8.85) | **17 more per 1,000** (from 7 more to 35 more) | ⨁⨁◯◯ Low^b,c^ | CRITICAL |

| **Cesarean Section** | | | | | | | | | | | | |
| --- | --- | --- | --- | --- | --- | --- | --- | --- | --- | --- | --- | --- |
| 10 | Non-RCTs | not serious | serious^a^ | not serious | not serious | none | 5275/10969 (48.1%) | 45300/104186 (43.5%) | **OR 1.44** (1.16 to 1.79) | **91 more per 1,000** (from 37 more to 145 more) | ⨁⨁⨁◯ Moderate^a^ | CRITICAL |
| **Intrauterine Growth Restriction (IUGR)** | | | | | | | | | | | | |
| 8 | Non-RCTs | not serious | serious^a^ | not serious | Serious^c^ | none | 123/498 (24.7%) | 98/473 (20.7%) | **OR 3.03** (1.16 to 7.91) | **46 more per 1,000** (from 37 more to 194 more) | ⨁⨁◯◯ Low^a,d^ | CRITICAL |
| **Live Birth** | | | | | | | | | | | | |
| 4 | Non-RCTs | not serious | serious^a^ | serious^b^ | serious^d^ | none | 269/478 (56.3%) | 478/743 (64.3%) | **OR 0.65** (0.41 to 1.02) | **104 fewer per 1,000** (from 218 fewer to 5 more) | ⨁◯◯◯ Very low^a,b,d^ | CRITICAL |
| **Neonatal Intensive Care Unit (NICU) Admission** | | | | | | | | | | | | |
| 5 | Non-RCTs | not serious | not serious | serious^b^ | serious^c^ | none | 82/707 (11.6%) | 223/5714 (3.9%) | **OR 2.20** (1.63 to 2.97) | **43 more per 1,000** (from 23 more to 69 more) | ⨁⨁◯◯ Low^b,c^ | CRITICAL |
| **Natural Vaginal Delivery** | | | | | | | | | | | | |
| 8 | Non-RCTs | not serious | serious^a^ | not serious | not serious | none | 3819/7587 (50.3%) | 45470/87781 (51.8%) | **OR 0.68** (0.51 to 0.91) | **96 fewer per 1,000** (from 164 fewer to 24 fewer) | ⨁⨁⨁◯ Moderate^a^ | CRITICAL |

**CI:** confidence interval; **RCT**: randomized controlled trials; **OR:** odds ratio

#### Explanations

a. Serious inconsistency since I2>60%. Downgraded.

b. Serious Indirectness since limited number of studies included. Downgraded.

c. Serious Imprecision since very wide CI. Downgraded.

d. Serious Imprecision since the meta-analysis result was not statistically significant. Downgraded.
